# Immunogenicity of the Monovalent Omicron XBB.1.5-Adapted BNT162b2 COVID-19 Vaccine in People Living with HIV (PLWH)

**DOI:** 10.3390/vaccines12070785

**Published:** 2024-07-17

**Authors:** Maxim Cherneha, Isabel Zydek, Peer Braß, Johannes Korth, Sarah Jansen, Stefan Esser, Christina B. Karsten, Folker Meyer, Ivana Kraiselburd, Ulf Dittmer, Monika Lindemann, Peter A. Horn, Oliver Witzke, Laura Thümmler, Adalbert Krawczyk

**Affiliations:** 1 Department of Infectious Diseases, West German Centre of Infectious Diseases, University Medicine Essen, University Hospital Essen, University Duisburg-Essen, 45147 Essen, Germany; maxim.cherneha@uk-essen.de (M.C.); isabel.zydek@uk-essen.de (I.Z.); peer.brass@uk-essen.de (P.B.); oliver.witzke@uk-essen.de (O.W.); laura.thuemmler@uk-essen.de (L.T.); 2 Department of Nephrology, University Medicine Essen, University Hospital Essen, University Duisburg-Essen, 45147 Essen, Germany; korth@dialyse-kortumpark.de; 3 Practice for Kidney Diseases, Dialysis and Apheresis, 44789 Bochum, Germany; 4 Institute for the Research on HIV and AIDS-Associated Diseases, University Medicine Essen, University Hospital Essen, University Duisburg-Essen, 45147 Essen, Germany; stefan.esser@uk-essen.de (S.E.); christina.karsten@uk-essen.de (C.B.K.); 5 Institute for Artificial Intelligence in Medicine, University Hospital Essen, University Duisburg-Essen, 45147 Essen, Germany; folker.meyer@uk-essen.de (F.M.); ivana.kraiselburd@uk-essen.de (I.K.); 6 Institute for Virology, University Medicine Essen, University Hospital Essen, University Duisburg-Essen, 45147 Essen, Germany; ulf.dittmer@uk-essen.de; 7 Institute for Transfusion Medicine, University Medicine Essen, University Hospital Essen, University Duisburg-Essen, 45147 Essen, Germany; monika.lindemann@uk-essen.de (M.L.); peter.horn@uk-essen.de (P.A.H.)

**Keywords:** COVID-19, PLWH, mRNA-Vaccine, SARS-CoV-2, immunity and immunocompromised

## Abstract

While SARS-CoV-2 has transitioned to an endemic phase, infections caused by newly emerged variants continue to result in severe, and sometimes fatal, outcomes or lead to long-term COVID-19 symptoms. Vulnerable populations, such as PLWH, face an elevated risk of severe illness. Emerging variants of SARS-CoV-2, including numerous Omicron subvariants, are increasingly associated with breakthrough infections. Adapting mRNA vaccines to these new variants may offer improved protection against Omicron for vulnerable individuals. In this study, we examined humoral and cellular immune responses before and after administering adapted booster vaccinations to PLWH, alongside a control group of healthy individuals. Four weeks following booster vaccination, both groups exhibited a significant increase in neutralizing antibodies and cellular immune responses. Notably, there was no significant difference in humoral immune response between PLWH and the healthy controls. Immune responses declined rapidly in both groups three months post vaccination. However, PLWH still showed significantly increased neutralizing antibody titers even after three months. These findings demonstrate the efficacy of the adapted vaccination regimen. The results suggest that regular booster immunizations may be necessary to sustain protective immunity.

## 1. Introduction

The COVID-19 pandemic, caused by the SARS-CoV-2, has presented unprecedented global health challenges, leading to a widespread deployment of vaccination campaigns aimed at reducing the pandemic’s impact [1].

During the COVID-19 pandemic, vaccination campaigns were pivotal in mitigating the spread of the virus and reducing morbidity and mortality rates, especially among vulnerable populations [2,3,4]. The mRNA vaccines have demonstrated significant efficacy in preventing severe disease outcomes [5,6]. In vulnerable patient cohorts, such as geriatric and immunocompromised individuals, including people living with HIV (PLWH), these vaccines have proven to be a cornerstone of public health strategy. Studies indicate that while the immunogenicity of mRNA vaccines may be slightly reduced in these populations, their effectiveness in preventing severe COVID-19 complications remains substantial, underscoring the importance of widespread vaccination efforts in these high-risk groups [7,8,9,10,11]. Notably, the emergence of new SARS-CoV-2 variants has raised serious concerns about the efficacy of the initially designed vaccines to protect from disease. Moreover, several studies have reported that vaccinated people get breakthrough infections [12,13,14].

Currently, Omicron sublineages continue to emerge: in October 2023, EG.5.1 was predominant globally, and since the end of November 2023, BA.2.86 and JN.1 have been expanding rapidly, with JN.1 being the predominant lineage in February 2024 in Germany [15]. In response to the continued evolution of Omicron sublineages, on 31 August 2023, the European Medicines Agency authorized an updated monovalent BNT162b2 vaccine encoding the viral spike protein of SARS-CoV-2 Omicron XBB.1.5 (Comirnaty Omicron XBB.1.5, Pfizer-BioNTech, New York, NY, USA) for use in individuals ≥6 months of age, based on the epidemiological predictions of the time [16]. In the following months, the first data on the immunogenicity and clinical efficacy of the updated vaccine were gathered worldwide [17,18]. However, data on immunocompromised populations are scarce. As the virus evolves, understanding the vaccine’s immunogenicity across diverse populations, especially among vulnerable groups such as PLWH, becomes paramount. PLWH represent a critical population in vaccine research due to their altered immune responses, which can affect vaccine efficacy [19,20,21].

HIV infection disrupts the immune system primarily by depleting CD4^+^ T cells [22]. The degree of immune compromise in HIV—often gauged by CD4^+^ T cell counts and viral load of HIV—can modulate vaccine responsiveness, with those having lower CD4^+^ T cell counts or unsuppressed viral loads potentially showing less robust cellular responses [11,23,24]. A systematic review and meta-analysis, which adjusted patient outcomes for age and sex, reported that HIV-positive COVID-19 patients were more likely to be hospitalized and had a higher risk of death [25].

This depletion, along with HIV-associated immune dysregulation, can impair both humoral and cellular immune responses to vaccines. Recent studies have shown that the humoral response to COVID-19 vaccination, in terms of antibody production, is surprisingly similar in PLWH compared to healthy controls [9,10,26], suggesting that PLWH can achieve comparable levels of seroconversion and antibody persistence after COVID-19 vaccination. However, these studies have also suggested a disparity in cellular immunogenicity, with PLWH often exhibiting weaker cellular responses to COVID-19 vaccines [9,27]. This differentiation in cellular response is crucial, as T cell responses are pivotal for long-term immunity and protection against severe COVID-19.

This nuanced understanding of vaccine-induced immunity in PLWH underscores the necessity for continued research to optimize vaccination strategies for this population, especially given the emergence of variants known for their heightened transmissibility and potential for immune evasion. The emergence of Omicron variants, known for their increased transmissibility and potential for immune escape, has intensified the need to investigate the adapted BNT162b2 vaccine’s efficacy in eliciting a robust immune response in this population. The aim of our study was to prospectively evaluate the immunogenicity of the monovalent Omicron XBB.1.5-adapted BNT162b2 COVID-19 vaccine among PLWH through an interventional clinical trial. We compared immune responses between PLWH and healthy controls at one and three months post vaccination. Furthermore, we measured neutralizing antibody titers and specific T cell responses against the D614G strain, Omicron BA.5 (BE.1.1), and XBB.1 (EG.5.1.1) sublineages. Our study seeks to provide valuable insights into the efficacy of the adapted vaccine in immunocompromised populations amidst the evolving pandemic landscape.

## 2. Materials and Methods

### 2.1. Study Cohort

This prospective, monocentric cohort study was conducted to evaluate the immunogenicity of the monovalent Comirnaty Omicron XBB.1.5 COVID-19 vaccine in people living with HIV (PLWH). The study population comprised patients registered at the outpatients’ clinic of the Department of Dermatology at the University of Medicine Essen who were recruited from September to December 2023. Inclusion criteria included individuals aged 18 years and older, diagnosed with HIV, and who were stable on antiretroviral therapy (ART) for at least six months prior to enrolment. Patients with a known history of COVID-19 vaccination received the Comirnaty Omicron BNT162b2 XBB.1.5 vaccine as a booster dose. Key exclusion criteria were acute or chronic infections other than HIV, vaccination with any other COVID-19 vaccine within the past 12 months, and known allergies to vaccine components. The cohort consisted of 27 men and 2 women with a median age of 56 years (range 33–77). The control cohort contained 14 healthcare workers from the University of Medicine Essen. The group consisted of six men and eight women with a median age of 32 years (range 26–58). These participants, presumed to have baseline immune competency, also received the Comirnaty Omicron XBB.1.5 vaccine as a booster dose. Upon recruitment, baseline data, including demographics, HIV viral load, CD4^+^ T cell count, and previous COVID-19 vaccination history, were collected for the PLWH cohort (Table 1). Similarly, baseline demographic data and previous COVID-19 vaccination history were collected for the medical worker cohort.

### 2.2. Study Design

In total, 65 PLWH were initially included in this study and were vaccinated with the Comirnaty Omicron XBB.1.5 vaccine as a booster dose. At time point t1, 29 participants were excluded due to not attending the appointment. At t2, seven participants were excluded. Finally, 29 PLWH were enrolled in this study. The cohort consisted of 27 men and 2 women with a median age of 56 years (range 33–77), and the control cohort contained 14 healthcare workers, 6 men and 8 women, from the University of Medicine Essen who were also vaccinated with the Comirnaty Omicron XBB.1.5 vaccine.

**Figure 1:**
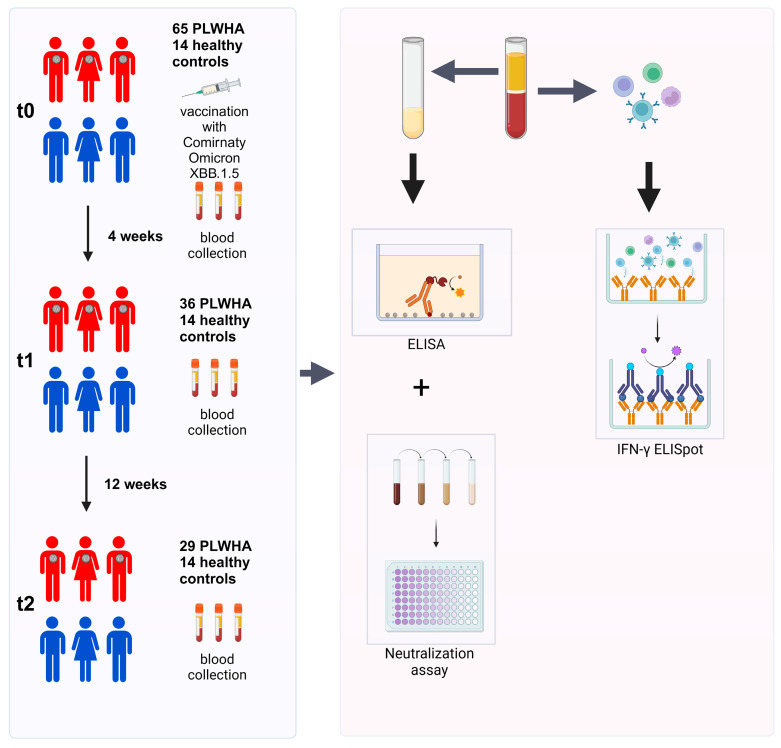
Study overview. Initially, 65 PLWH and 14 healthy controls were enrolled in this study. Out of these, 36 PLWH did not complete the study and were excluded from the analysis. Blood samples were collected before the first immunization (t0), as well as four (t1) and 12 weeks (t2) after immunization. PBMCs were used to investigate the cellular immune response by IFN-γ ELISpot, while sera were utilized for analyzing the humoral immune response by ELISA and a cell-culture-based neutralization assay.

Blood samples were collected at t0 (on the day of the booster immunization), t1 (four weeks after booster immunization), and t2 (12 weeks after booster immunization). Sera were used to determine the humoral immune response by ELISA and a cell-culture-based neutralization assay. PBMCs were isolated to analyze the cellular immune response by IFN-γ ELISpot (Figure 1).

### 2.3. Cells and Viruses

A549-AT cells are stably transfected cells that have been engineered to overexpress the angiotensin-I-converting enzyme 2 (ACE2) receptor and the cellular transmembrane protease serine 2 (TMPRSS2). This modification enhances cytopathic effects (CPE) and increases susceptibility to SARS-CoV-2 [28]. A549-AT cells were cultured in Minimum Essential Medium (MEM) supplemented with 10% fetal calf serum (FCS), 4 mM L-glutamine, 100 IU/mL penicillin, and 100 μg/mL streptomycin at 37 °C and 5% CO_2_. As previously described, the clinical SARS-CoV-2 isolates D614G (wild-type), Omicron BA.5, and Omicron XBB.1 were obtained from nasopharyngeal swabs of COVID-19 patients hospitalized at the University Hospital Essen [29,30]. The viruses were propagated on A549-AT cells and stored at −80 °C. Viral titers were determined using A549-AT cells by a standard endpoint dilution assay and calculated as 50% tissue culture infective dose (TCID50)/mL. SARS-CoV-2 whole-genome libraries were obtained with the EasySeq™ SARS-CoV-2 Whole Genome NGS Sequencing kit (Nimagen, Nijmegen, The Netherlands) as previously described [12]. The specific variants were identified through sequence analysis using the WHO list of variants of concern [31].

### 2.4. ELISA

To detect SARS-CoV-2-specific antibodies, a CE-marked anti-SARS-CoV-2 IgG quantitative ELISA (anti-SARS-CoV-2-QuantiVac-ELISA, Euroimmun, Lübeck, Germany) was used. The ELISA was performed according to the manufacturer’s instructions at a dilution from 1:2000 to 1:4000 of the sera. Plates were coated with wild-type recombinant SARS-CoV-2 spike protein (S1 domain). Results were measured as binding antibody units (BAU)/mL.

### 2.5. Cell-Culture-Based Neutralization Assay

To assess the neutralizing antibody serum titers, a standard endpoint dilution assay was used, as described previously [7]. Briefly, serial dilutions (1:20 to 1:2560) of the respective sera were incubated with 100 TCID_50_ of SARS-CoV-2 D614G (wild-type), Omicron BA.5, or Omicron XBB.1 for one hour at 37 °C, 5% CO_2_. Afterward, the dilutions were added to confluent A549-AT cells in 96-well microtiter plates and incubated for 3 days at 37 °C, 5% CO_2_. Next, the cells were stained with crystal violet (Roth, Karlsruhe, Germany) solved in 20% methanol (Merck, Darmstadt, Germany) and analyzed for the presence of cytopathic effects (CPE) by light microscopy. The neutralizing titer was defined as the reciprocal of the highest serum dilution at which CPE was not observed in any of the three test wells.

### 2.6. In House ELISpot

To assess SARS-CoV-2-specific cellular immunity, we employed an in-house IFN-γ enzyme-linked ImmunoSpot (ELISpot) assay, as previously described [1]. ELISpot stripes containing PVDF membranes (MilliporeSigma MultiScreen HTS; Thermo Fisher Scientific, Schwerte, Germany) were activated with 50 μL of 35% ethanol for 10 s. After washing with distilled water, stripes were coated with 60 μL of monoclonal antibodies against IFN-γ (10 μg/mL of clone 1-D1K; Mabtech, Nacka, Sweden) for 3 h at 37 °C, 5% CO_2_. Thereafter, stripes were washed and blocked with 150 μL AIM-V^®^ (Thermo Fisher Scientific) for 30 min at 37 °C, 5% CO_2_. After blocking, AIM-V^®^ was discarded and 250,000 peripheral blood mononuclear cells (PBMCs) were grown in duplicates in the presence or absence of either SARS-CoV-2 peptide pool, Omicron BA.4/BA.5 full-length SARS-CoV-2 peptide pool, or XBB.1.5.X Omicron full-length SARS-CoV-2 peptide pool (600 pmol/mL of each peptide, Peptide & Elephants, Henningsdorf, Germany) for 19 h, 37 °C, 5% CO_2_. Thereafter, the stripes were washed and incubated with 50 μL of the alkaline phosphatase-conjugated monoclonal antibody against IFN-γ (clone 7-B6–1, Mabtech), diluted 1:200 with phosphate-buffered saline plus 0.5% bovine serum albumin for 1 h at room temperature. After further washing, 50 μL of nitro blue tetrazolium/5-bromo-4-chloro-3-indolyl-phosphate was added for 7 min until purple spots appeared. Spot numbers were analyzed by an ELISpot reader (AID Fluorospot, Autoimmun Diagnostika GmbH, Strassberg, Germany). Mean values of duplicate cell cultures were considered. We determined SARS-CoV-2-specific spots by spot increment, defined as stimulated minus nonstimulated values. Stimulated spot numbers > 3-fold higher than negative (unstimulated) controls were considered positive.

### 2.7. Ethics

Prior to study initiation, all study participants provided informed consent, and the study protocol was reviewed and approved by the Ethics Committee of the University Hospital Essen (approval numbers: 20-9753-BO and 20-9208-BO), in accordance with the 1964 Declaration of Helsinki, amended in 2013.

### 2.8. Graphics and Statistical Analyses

Statistical analyses were conducted using GraphPad Prism 10.2.2 software (GraphPad Software, San Diego, CA, USA). Comparisons between PLWH and the control group were made using the Mann–Whitney test. Friedman test with Dunn’s multiple comparison test were used to compare the responses at different time points within one group. One-sided *p*-values < 0.05 were considered significant. Schematic illustrations were created using BioRender.

## 3. Results

### 3.1. SARS-CoV-2-Specific ELISA Antibody Concentrations

**Figure 2:**
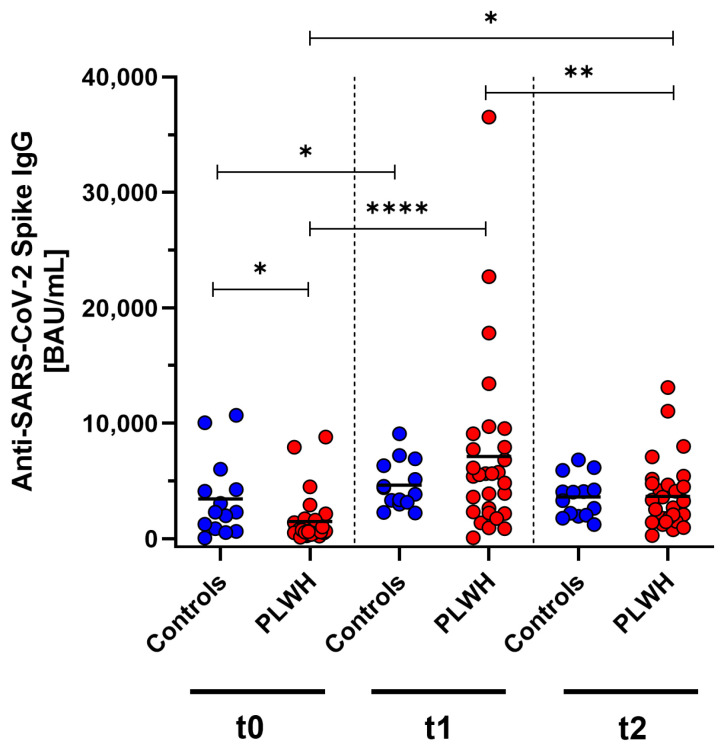
Concentration of anti-SARS-CoV-2 Spike IgG before (t0), one month (t1), and three months (t2) after Comirnaty Omicron XBB.1.5 vaccination in PLWH and healthy controls. Blue circles show data of healthy controls, while red circles indicate data of PLWH. Friedman test with Dunn’s multiple comparison test were used to compare the responses at different time points within one group, while one-tailed Mann–Whitney test was used to compare responses between the cohorts (* *p* < 0.05, ** *p* < 0.01, **** *p* < 0.0001). Median is represented by horizontal lines.

SARS-CoV-2-specific antibody concentrations were measured in PLWH and a control group of immunocompetent medical workers at baseline, one month post vaccination, and three months post vaccination using ELISA. Initially, before the vaccination, the antibody concentrations were significantly lower in PLWH compared with the control group (*p* = 0.013). One month after receiving the Comirnaty Omicron XBB.1.5 vaccine booster dose, both cohorts showed a significant increase (controls: *p* = 0.013, PLWH: *p* < 0.0001) compared with baseline. In the control group, the antibody concentrations also increased one month after vaccination, but this did not reach significance. Three months following vaccination, a significant decline in antibody levels was observed in PLWH (*p* = 0.0031). Despite this reduction, the antibody concentrations remained significantly elevated above the prevaccination baseline in PLWH (*p* = 0.011). The decrease in concentrations was seen in both the PLWH and the control group (Figure 2).

### 3.2. PLWH Develop a Comparable Humoral Immune Response to Healthy Controls

**Figure 3:**
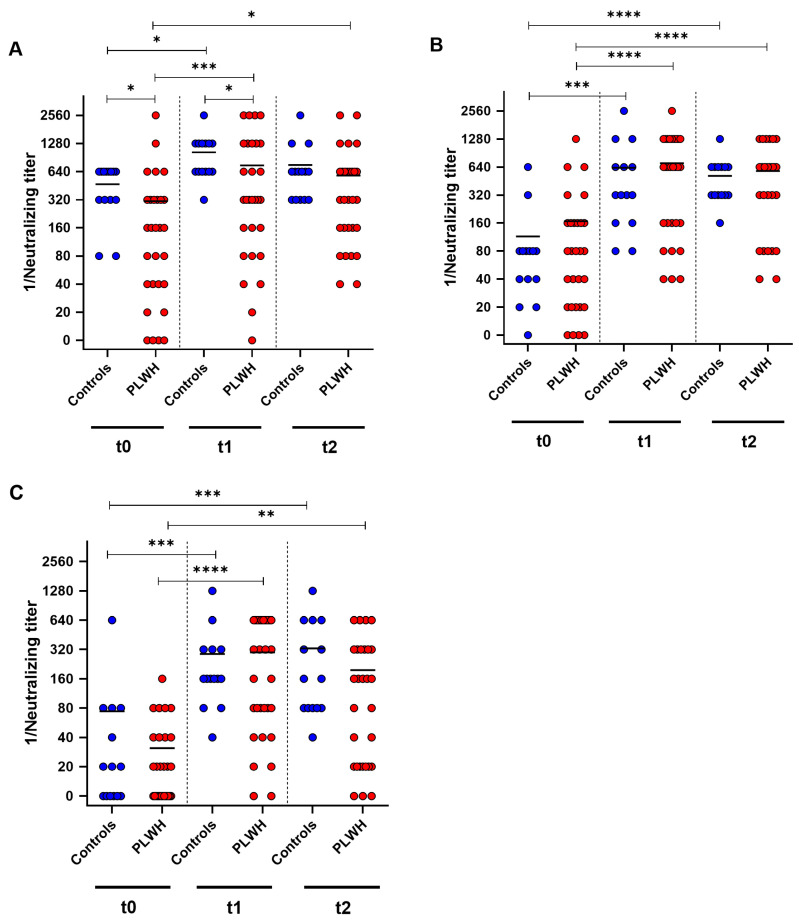
SARS-CoV-2-specific neutralizing antibody titers before (t0), one month (t1), and three months (t2) after Comirnaty Omicron XBB.1.5 vaccination in PLWH and healthy controls. The reciprocal of the neutralizing anti-SARS-CoV-2 antibody titer is presented for (**A**) D614G (wild-type), (**B**) Omicron BA.5, and (**C**) Omicron XBB.1.5. Blue circles show data of healthy controls, while red circles indicate data of PLWH. Friedman test with Dunn’s multiple comparison test were utilized to compare the responses at different time points within each group, whereas a one-tailed Mann–Whitney test was employed to compare responses between the cohorts (* *p* < 0.05, ** *p* < 0.01, *** *p* < 0.001, **** *p* < 0.0001). Median is represented by horizontal lines.

To further analyze the humoral immune response towards the Comirnaty Omicron XBB.1.5 vaccine, neutralizing antibody titers against the SARS-CoV-2 variants D614G, Omicron BA.5 and Omicron XXB.1 were determined by cell-culture-based neutralization assay before vaccination, and one and three months after vaccination. Both groups showed a significant increase in neutralizing antibodies against D614G one month after booster immunization (control: *p* = 0.014, PLWH: *p* = 0.0002) (Figure 3A). PLWH displayed significantly lower neutralizing antibody titers at baseline and one month post vaccination compared with controls (baseline: *p* = 0.0037, one month: *p* = 0.027). Furthermore, PLWH had significantly higher neutralizing titers three months after vaccination compared with baseline (*p* = 0.014) (Figure 3A).

Against Omicron BA.5, both groups demonstrated increased neutralizing antibody titers one month after the booster vaccination (control: *p* = 0.0005, PLWH: *p* < 0.0001). Three months after vaccination, both groups had significantly higher titers of neutralizing antibodies compared with baseline (control: *p* < 0.0001, PLWH: *p* < 0.0001). PLWH and healthy controls exhibited comparable levels of neutralizing antibodies against Omicron BA.5 at all three time points of evaluation (Figure 3B). PLWH and healthy controls exhibited a significant increase in humoral immune response towards Omicron XBB.1 one month after vaccination (controls: *p* = 0.0002, PLWH: *p* < 0.0001). Both cohorts had significantly higher neutralizing antibody titers three months post vaccination compared with baseline (control: *p* = 0.0007, PLWH: *p* = 0.0019) (Figure 3C).

### 3.3. SARS-CoV-2-Specific T Cell Response

**Figure 4:**
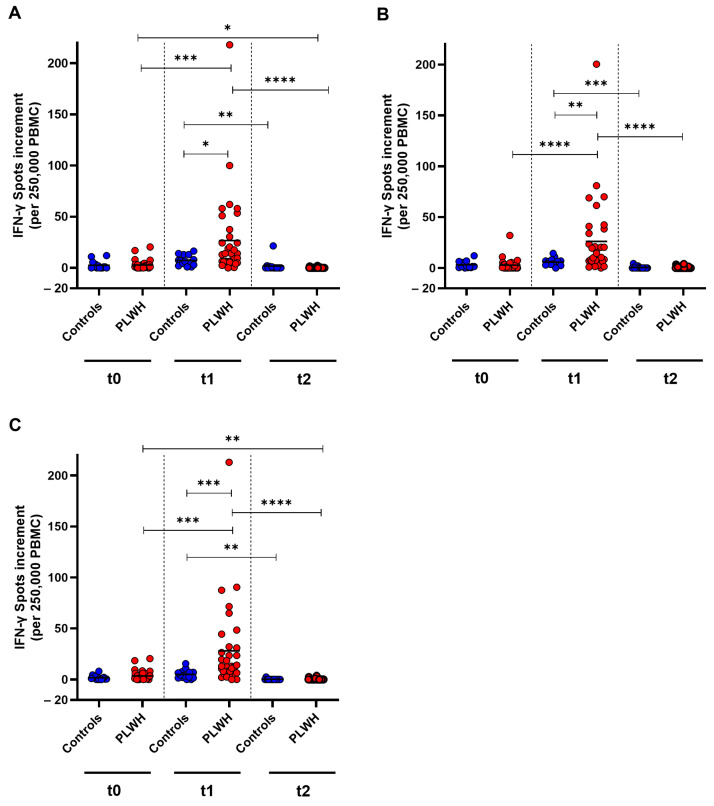
SARS-CoV-2-specific cellular immune response before, one month, and three months after Comirnaty Omicron XBB.1.5 vaccination in PLWH and healthy controls. The IFN-γ spot increments for (**A**) D614G (wild-type), (**B**) Omicron BA.5, and (**C**) Omicron XBB.1.5 are depicted. Data from healthy controls are represented by blue circles, while data from PLWH are indicated by red circles. Friedman test with Dunn’s multiple comparison test were utilized to compare responses at different time points within each group, while a one-tailed Mann–Whitney test was employed to compare responses between the cohorts (* *p* < 0.05, ** *p* < 0.01, *** *p* < 0.001, **** *p* < 0.0001). Median is represented by horizontal lines.

To examine the cellular immune response towards SARS-CoV-2 wild-type, as well as Omicron BA.5 and XBB.1.5 before and after booster vaccination, an IFN-γ ELISpot was used. For the wild-type strain, a significant increase in T cell response was detected in PLWH one month post vaccination (*p* = 0.0002). However, the cellular immune response decreased significantly in both cohorts three months after vaccination compared with the spot increment observed one month post vaccination (control: *p* = 0.003, PLWH: *p* < 0.0001). PLWH had significantly higher spot increments compared with healthy controls one month after vaccination (*p* = 0.045). The spot increment in PLWH was significantly lower after three months compared with baseline (*p* = 0.010) (Figure 4A).

Against Omicron BA.5, PLWH demonstrated a significant increase in cellular immune response one month after vaccination, whereas healthy controls did not exhibit a significant increase (*p* < 0.0001). The IFN-γ spot increment decreased significantly three months post vaccination in PLWH (*p* < 0.0001) compared with the spot increment one month after vaccination. One month after vaccination, PLWH had significantly higher spot increments compared with healthy controls (*p* = 0.0023) (Figure 4B).

The cellular immune response towards Omicron XBB.1.5 increased significantly in PLWH one month after vaccination (*p* < 0.0001) and then decreased significantly in both cohorts within two months (controls: *p* = 0.0028, PLWH: *p* < 0.0001). The spot increment three months post vaccination was significantly lower in PLWH compared with baseline (*p* = 0.0089). A significant difference between the groups was only detected one month after vaccination, where PLWH had significantly higher spot increments compared with healthy controls (*p* = 0.0003) (Figure 4C).

### 3.4. Humoral and Cellular Immune Responses towards Different SARS-CoV-2 Variants

**Figure 5:**
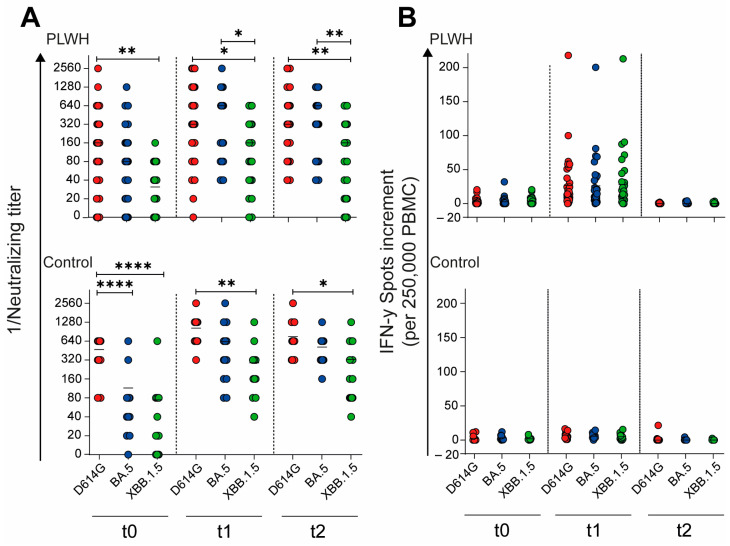
SARS-CoV-2-specific humoral and cellular immune response before, one month, and three months after Comirnaty Omicron XBB.1.5 vaccination in PLWH and healthy controls. (**A**) The reciprocal of the neutralizing anti-SARS-CoV-2 antibody titers and (**B**) the IFN-γ spot increments in PLWH and healthy controls. Red circles show data of D614G, blue circles show data of Omicron BA.5, and green circles show data of Omicron XXB.1.5. Ordinary one-way ANOVA was utilized to compare the responses at different time points against different SARS-CoV-2 variants (* *p* < 0.05, ** *p* < 0.01, **** *p* < 0.0001). Median is represented by horizontal lines.

To evaluate the impact of the adapted vaccination on the neutralizing capacity of antibodies against different SARS-CoV-2 variants, we compared the neutralizing antibody titers across time points and variants. In the cohort of PLWH, the neutralizing antibody titers against the D614G variant were significantly higher compared with those against Omicron XBB.1.5 at all time points: baseline (t0: *p* = 0.0058), 4 weeks post vaccination (t1: *p* = 0.021), and 12 weeks post vaccination (t2: *p* = 0.0067), as shown in Figure 5A. Similarly, in the control cohort, the neutralizing titers for D614G were significantly higher than those for both Omicron BA.5 and XBB.1.5 (*p* < 0.0001) at baseline (Figure 5A). This significant difference persisted for D614G compared with Omicron XBB.1.5 at both post-vaccination time points: 4 weeks (t1: *p* = 0.0025) and 12 weeks (t2: *p* = 0.037) after vaccination. These results suggest that while the adapted vaccination elicited a robust antibody response, the neutralizing capacity remained variant-dependent, with consistently higher titers against the D614G variant compared with newer Omicron subvariants across both cohorts and all time points.

Building upon our analysis of the humoral immune response, we further examined the impact of the Omicron XBB.1.5-adapted vaccine on the cellular immune response toward different SARS-CoV-2 variants. Unlike the variant-dependent differences observed in neutralizing antibody titers, the cellular immune response showed no significant differences across variants, either in PLWH or in healthy controls (Figure 5B). This finding suggests that while the neutralizing antibody response remains variant-specific, the cellular immune response elicited by the adapted vaccine appears to be more broadly reactive across the tested SARS-CoV-2 variants. These results highlight the importance of considering both humoral and cellular immunity when evaluating the effectiveness of adapted COVID-19 vaccines, particularly in diverse populations such as PLWH and healthy individuals.

## 4. Discussion

The neutralizing antibodies produced by vaccination against the ancestral strain of SARS-CoV-2 exhibit reduced effectiveness against newly emerged variants, particularly the Omicron variant and its sublineages [32,33]. The resulting increase in breakthrough infections can lead to severe outcomes, especially for vulnerable individuals, such as PLWH. The vaccine adapted by Pfizer-BioNTech was intended to offer protection against the Omicron variants BA.5 and the sublineage XBB.1.5.

In the present study, the humoral and cellular immune responses to the Omicron XBB.1.5-adapted BNT162b2 vaccine were examined in 29 people living with HIV and 14 healthy controls. Assessments were conducted before vaccination, as well as one and three months post vaccination. The humoral immune response was analyzed using anti-SARS-CoV-2 spike IgG and a cell-culture-based neutralization assay. The cellular immune response was evaluated using the IFN-γ ELISpot assay.

Before the first vaccination, PLWH showed lower SARS-CoV-2-specific ELISA antibody concentrations than healthy controls. A possible explanation for this is a generally reduced immunity in PLWH undergoing antiretroviral therapy [34]. Moreover, it is worth considering that the healthy control group comprised medical professionals, whose occupation might have resulted in elevated baseline antibody concentrations. This heightened immunity could stem from their increased exposure to SARS-CoV-2 and possibly other coronaviruses. However, our ability to accurately assess infection rates is limited due to the discontinuation of mandatory SARS-CoV-2 testing for medical professionals prior to the commencement of our study, which represents a limitation of the study. Despite the initial differences in baseline antibody concentrations, PLWH exhibited post-vaccination antibody concentrations comparable to those observed in the healthy control group. Four weeks after vaccination, PLWH demonstrated a notable rise in SARS-CoV-2-specific ELISA antibody concentrations. This result aligns with previous studies that examined the humoral immune response in older individuals and hemodialysis patients following booster vaccinations against Omicron sublineages [35,36]. Healthy controls exhibited only a slight increase in SARS-CoV-2-specific ELISA antibody concentrations, which contrasts with a previous study on the immunogenicity of the adapted vaccine [18]. The increase may not have been significant due to the higher baseline antibody concentrations. Three months after vaccination, PLWH showed a significant decrease in IgG antibodies, whereas healthy controls exhibited only a slight decrease. This is consistent with prior studies, which demonstrated a decrease in SARS-CoV-2-specific ELISA antibody concentrations six months after vaccination in both healthy controls and PLWH [37,38].

Both cohorts exhibited a significant increase in neutralizing antibodies four weeks after vaccination with the monovalent Omicron XBB.1.5-adapted vaccine against SARS-CoV-2 wild-type (D614G), Omicron BA.5, and Omicron XBB.1. These findings are consistent with a previous Italian study, which showed an increased humoral immune response against wild type (D614G), BA.5, BQ1.1, and XBB.1, 15 days after vaccinating PLWH with an mRNA bivalent vaccine [39]. Comparable results were achieved in the vulnerable group of hemodialysis patients after vaccination with the adapted mRNA vaccine [40]. Three months after vaccination, neutralizing antibody levels decreased in both cohorts. PLWH showed slightly decreased neutralizing antibody titers against Omicron XBB.1, while the titers against the wild type (D614G) and Omicron BA.5 remained comparable to those in healthy controls. Previous studies have shown similar results three months after mono- or bivalent vaccination against SARS-CoV-2 Omicron variants and sublineages in different cohorts [41,42,43,44].

For cellular immune response, a significant increase in IFN-γ producing cells was observed in PLWH for all tested SARS-CoV-2 variants, while healthy controls showed only a slight increase. This finding contrasts with another study, where the cellular immune response did not increase after bivalent mRNA vaccination [45].

A possible reason for this significant increase in cellular response might be the chronic immune activation characteristic of HIV infection, which could enhance the responsiveness of T cells to vaccination [34]. HIV infection leads to continuous immune activation, even when viral replication is controlled by antiretroviral therapy (ART). This chronic immune activation affects both the quantity and quality of T cells. Despite the immunodeficiency associated with HIV, the remaining T cells can become hyper-responsive due to ongoing activation signals. This persistent activation can prime the immune system for a more robust response, resulting in increased IFN-γ production by T cells. Chronic activation involves various immune components and cytokines, such as tumor necrosis factor (TNF), interleukins (IL-6, IL-8), and type I interferons (IFN-α, IFN-β). The underlying studies indicate that this state of persistent activation results in an upregulated immune environment that can prime T cells for heightened responses to subsequent stimuli, such as vaccination [34,46]. The causes of chronic immune activation in HIV are multifactorial. They include direct effects of the virus, such as the presence of viral proteins and nucleic acids, as well as indirect effects, such as microbial translocation due to gastrointestinal tract damage. This continuous exposure to immune stimuli keeps the immune system in a state of heightened alertness, which can result in exaggerated responses when new antigens are introduced [47]. Another example of this effect is a study comparing IFN-γ production between HIV-infected individuals and healthy controls using the QuantiFERON Monitor (QFM) assay. The study found that IFN-γ production was significantly higher in HIV-infected participants than in healthy controls, particularly among those HIV-infected participants with a recovered CD4^+^ T cell count > 350/μL [48].

These conditions might lead to a heightened state of immune readiness, resulting in a more pronounced T cell response upon vaccination. This might have also led to increased exposure to SARS-CoV-2, which could have enhanced the T cell response, similar to reported findings of increased VZV-specific T cell responses in individuals with recent VZV reactivations [37]. Furthermore, it is surprising that T cell responses showed a significant decrease below baseline three months after vaccination in both groups. One possible explanation might be the timing of this study in the context of the development of the COVID-19 pandemic in Germany. Most participants were enrolled in this study in November 2023, with the t2 blood drawn in January 2024. While COVID-19 cases had been increasing until December, case numbers showed a sudden drop at the end of December and the beginning of January [49]. This decrease in circulating virus might explain a drop in measurable cellular immunity in peripheral T cells of both groups.

However, several limitations of this study should be acknowledged. First, the sample size was relatively small, potentially limiting the generalizability of our findings. Larger studies are needed to confirm the observed immune responses and to better understand the variability within the PLWH population. Second, while we included a control group of healthy medical workers, there may be other confounding factors, such as age, sex, and differences in occupational exposure to SARS-CoV-2 and other coronaviruses, which could influence baseline immunity and vaccine responses. Additionally, there was a significant age difference between the two study groups, which may have impacted the results. Looking ahead, as the virus continues to evolve, ongoing evaluation of vaccine efficacy in immunocompromised populations will be crucial to ensure sustained protection for vulnerable groups.

## 5. Conclusions

In conclusion, our study provides valuable insights into the immunogenicity of the Omicron XBB.1.5-adapted BNT162b2 vaccine in PLWH, demonstrating robust immune responses and highlighting the need for tailored vaccination strategies to maintain protective immunity. These findings contribute to the growing body of evidence supporting the efficacy of COVID-19 vaccines in immunocompromised populations and underscore the importance of regular booster doses to sustain immunity.

## Figures and Tables

**Table 1:** Patients’ characteristics.

Variable	Study Inclusion	3 Months Post Vaccination
Number [n]	29	
Male sex [n]	27 (93.1%)	
Age [yrs.], median (IQR)	56.7 (53.3; 61.1)	
Time on HIV Therapy [yrs.], median (IQR)	15 (10; 23)	
Antiretroviral therapy		
Bictegravire/Tenofovir alafenamide fumarate/Emtricitabine	11 (37.93%)	
Dolutegravir/Lamivudine	5 (17.24%)	
Rilpivirine/Emtricitabine/Tenofovir alafenamide fumarate	5 (17.24)	
Abacavir/Lamivudine/Dolutegravir	4 (13.79%)	
Other	4 (13.79%)	
Clinical HIV stage [n]		
A	12 (41.38%)	
B	11 (37.93%)	
C	6 (20.69%)	
CD4^+^ T cell count		
>500/μL	23 (79.31%)	22 (75,86%)
200–500/μL	5 (17.24%)	7 (24.14%)
<200/μL	1 (3.44%)	
Leucocytes [n/μL], median (IQR) normal range: 3600–9200	6760 (5390; 9330)	6390 (5493; 8703)
Lymphocytes [n/μL], median (IQR) normal range: 1000–3400	2440 (1950; 3210)	2225 (1718; 2700)
CD4^+^ T cells [n/μL], median (IQR) normal range: 300–1400	746 (583; 943)	677 (551; 922)
B cells [n/μL], median (IQR) normal range: 100–500	220 (168; 296)	187 (133; 287)
HIV viral load [RNA cop./mL]		
>20	1 (3.44%)	1 (3.44%)
<20	28 (96.55%)	28 (96.55%)
Count of confirmed SARS-CoV-2 antigen exposure (vaccination/infection)		
7	1 (3.44%)	
6	2 (6.9%)	
5	7 (24.14%)	
4	12/41.38%)	
3	6 (20.69%)	
1	1 (3.44%)	

## Data Availability

The data presented in this study are available on request from the corresponding author.
